# Data on records of temperature and relative humidity in a building model with green facade systems

**DOI:** 10.1016/j.dib.2019.104896

**Published:** 2019-11-27

**Authors:** Ratih Widiastuti, Juliana Zaini, Wahyu Caesarendra

**Affiliations:** a Faculty of Integrated Technologies, Universiti Brunei Darussalam, Jalan Tungku Link, Gadong, BE1410, Brunei Darussalam; b Department of Architectural Design, Vocational School, Diponegoro University, Semarang 50275, Indonesia

**Keywords:** Building model, Field data measurement, Green facade, Space temperature, Surface temperature, Relative humidity

## Abstract

Reducing cooling load is one of the important aspects to increase energy saving in the building. This paper presents the data record of temperatures (surface and space temperatures) and relative humidities in a building model integrated with green facade systems. The research was conducted in a tropical climate country. There were three types of green facade systems that act as the main parameter: 0%; 50%; and 90%. The preliminary studies were conducted at the Architecture Department of Diponegoro University in Semarang (Indonesia) and the data obtained is presented in this paper. The dataset was collected through field data measurement of green facade systems attached on the facade of the building model. The building model was observed during sunny days. The data presented in this article are related to the research article entitled Observation to building thermal characteristic of green façade model based on various leaves covered area [1].

**Table:** Specifications Table

Subject	Construction & building technology
Specific subject area	Thermal performance of building with green facade systems to minimize the cooling load inside building
Type of data	Table, text file
How data were acquired	Surface temperatures, space temperatures, and relative humidities were collected through field data measurement. Infrared surface temperature was used to measure interior and exterior surface temperatures. Hygro thermometer was used to measure space temperatures and relative humidities. Outdoor environmental data were acquired from the Maritime Meteorology Station of Semarang City [2].
Data format	Raw
Experimental factors	Due to limitation of building model, the experiment of green facades was conducted alternately and the dataset for each green facade type was acquired on different days. The local weather condition was considered. During field data measurement the local weather conditions were recorded relatively similar.
Experimental features	Three type of green facade systems with different leaves coverage area (stated in %) was attached on the building model scaled 1 m × 1 m x 1 m. Due to limitation of building model, the object study was monitored in different time during sunny days. Experiment 1 (green facade 0%) on 10th December 2013, experiment 2 (green facade 50%) on 13th December 2013 and experiment 3 (green facade 90%) on 16th December 2013. This paper presents in detail data collected on 10th December to 13th December.
Data source location	Architecture Department of Diponegoro University in Semarang (Indonesia). 7°03′05.9″S, 110°26′18.0″E
Data accessibility	Data are available within this article.
Related research article	Widiastuti, R, Caesarendra, W, Zaini, J. Observation to building thermal characteristic of green façade model based on various leaves covered area. Buildings 2019 9: 75. (DOI: https://doi.org/10.3390/buildings9030075) [1]

**Table:** 

**Value of the Data** • The data presented in this paper provides researchers the current research information related to thermal effect of green facade technologies in a building. • The dataset in this paper will assist researchers to evaluate further the coverage area of leaves on how it will affect the thermal performance of green facade. • The data will assist researchers to examine further building energy performance of the building with green facade systems or other vertical greenery systems. • The data given will assist researchers, practicing architects, and practicing engineers to consider and implement greenery aspects in building constructions as a strategy for energy saving.

## 1 Data

**Fig. 1:**
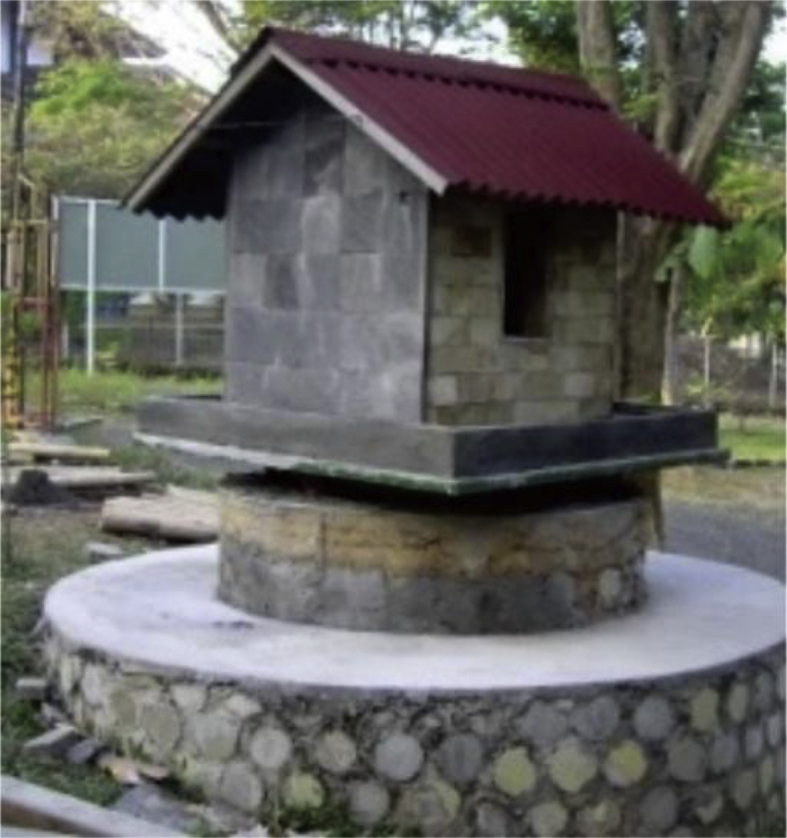
Building model for experiment of green facade [1].

The dataset contains raw data corresponded to the surface temperatures, space temperatures, and relative humidities in a building model with green facade systems located in Architecture Department of Diponegoro University in Semarang (Indonesia), can be seen in Fig. 1 and Fig. 3. Field data measurements were conducted during sunny days on 10th December 2013, 13th December 2013, and 16th December 2013. As for weather data were acquired from the Maritime Meteorology Station of Semarang City [2]. In this research, there are three types of green facade systems based on the percentage of leaves coverage area: 0%; 50%; and 100%, can be seen in Fig. 3. Table 1, Table 2 showed the dataset of surface temperatures, space temperatures and relative humidities in the each of green facade system, respectively.

**Table 1:** Surface temperature dataset of experiment 1, 2, and 3 on 10th, 13th, and 16th December 2013.

Time	10/12/2013 (experiment 1)		13/12/2013 (experiment 2)		16/12/2013 (experiment 3)	
	T_si_°	T_se_°	T_si_°	T_se_°	T_si_°	T_se_°
6:00	28.0	26.6	23.5	24.9	22.6	21.6
7:00	28.3	27.4	24.4	27.1	22.9	22.3
8:00	29.0	30.7	24.7	28.4	22.6	22.3
9:00	28.5	31.5	24.2	30.2	22.4	25.3
10:00	28.4	32.8	23.2	31.4	23.4	25.8
11:00	29.0	33.9	23.3	29.1	23.4	25.5
12:00	29.9	35.6	24.2	27.1	22.6	25.4
13:00	30.5	35.6	23.3	25.0	22.0	25.5
14:00	31.3	35.7	24.8	25.5	22.0	25.5
15:00	31.4	35.4	24.5	24.2	22.1	25.0
16:00	31.8	34.7	24.1	23.7	22.6	24.0
17:00	31.8	34.2	25.4	24.2	22.6	23.8
18:00	31.8	33.4	25.2	23.4	22.1	22.6
19:00	32.2	32.9	23.8	22.5	21.1	21.3
20:00	31.3	30.0	23.9	21.0	20.7	21.0
21:00	31.5	29.0	24.3	21.6	20.7	20.7
22:00	31.2	28.9	22.6	21.1	21.6	21.5
23:00	30.9	28.7	22.2	20.9	25.7	23.3
00:00	30.4	28.4	22.6	21.8	25.5	23.1
1:00	30.4	28.5	21.6	21.0	24.9	23.1
2:00	29.9	28.1	22.5	21.2	24.4	22.5
3:00	29.8	27.8	21.6	20.3	24.4	22.2
4:00	29.2	27.0	22.1	21.7	24.1	22.0
5:00	28.8	26.9	22.8	23.1	24.3	22.1

T_si_: interior surface temperature, T_se_: exterior surface temperature.

**Table 2:** Space temperature dataset of experiment 1, 2, and 3 on 10th, 13th, and 16th December 2013.

Time	10/12/2013 (experiment 1)		13/12/2013 (experiment 2)		16/12/2013 (experiment 3)	
	T_in_°	T_o_°	T_in_°	T_o_°	T_in_°	T_o_°
6:00	26.5	29.5	24.5	25.0	25.1	23.0
7:00	26.6	29.8	24.7	27.0	24.2	24.0
8:00	26.4	30.5	24.8	30.0	24.3	24.0
9:00	26.4	30.0	25.2	30.0	24.3	24.0
10:00	26.4	29.9	25.6	30.0	24.3	23.0
11:00	26.2	30.5	26.0	30.0	24.3	23.0
12:00	26.2	31.4	26.0	26.0	24.3	24.0
13:00	26.5	32.0	26.0	26.0	24.4	23.0
14:00	26.5	32.8	26.0	26.0	24.7	24.0
15:00	26.5	32.9	26.0	26.0	24.8	24.0
16:00	27.0	33.3	25.8	26.0	24.8	24.0
17:00	26.5	33.3	25.8	26.0	24.8	24.0
18:00	26.5	33.3	25.6	25.0	24.8	24.0
19:00	26.5	33.7	25.6	25.0	24.8	26.0
20:00	27.0	32.8	25.3	25.0	24.8	27.0
21:00	26.5	33.0	25.3	25.0	24.8	27.0
22:00	26.5	32.7	25.3	25.0	24.8	26.0
23:00	26.0	32.4	25.2	23.0	24.8	28.0
00:00	26.0	31.9	25.1	23.5	24.7	28.0
1:00	26.0	31.9	25.1	24.0	24.7	27.0
2:00	26.0	31.4	25.1	23.5	24.8	26.0
3:00	26.0	31.3	24.8	23.0	24.8	26.0
4:00	26.0	30.7	24.8	23.0	24.7	26.0
5:00	26.0	30.3	24.6	24.0	24.7	26.0

T_in_: indoor temperature; T_o_: outdoor temperature.

Table 1 showed dataset of surface temperatures for green facades with 0%, 50%, and 90% leaves coverage area. The average of surface temperatures on the experiment 1 with 0% leaves coverage area was 31.0 °C for exterior surface and 30.2 °C for interior surface. The maximum of interior surface temperature was 32.2 °C and occurred at 19:00 hr. The minimum of interior surface temperature was 28.0 °C and occurred at 06:00 hr. The maximum of exterior surface temperature was 35.7 °C and occurred at 14:00 hr. The minimum of exterior surface temperature was 26.6 °C and occurred at 06:00 hr.

The average of surface temperatures on the experiment 2 with 50% leaves coverage area was 24.2 °C for exterior surface and 23.5 °C for interior surface. The maximum of interior surface temperature was 25.4 °C and occurred at 17:00 hr. The minimum of interior surface temperature was 21.6 °C and occurred at 01:00 hr and 03:00 hr. The maximum of exterior surface temperature was 31.4 °C and occurred at 10:00 hr. The minimum of exterior surface temperature was 20.3 °C and occurred at 03:00 hr.

The average of surface temperatures on the experiment 3 with 90% leaves coverage area was 23.0 °C for interior surface and 23.2 °C for exterior surface. The maximum of interior surface temperature was 25.7 °C and occurred at 23:00 hr. The minimum of interior surface temperature was 20.7 °C and occured from 20:00 hr to 21:00 hr. The maximum of exterior surface temperature was 25.8 °C and occured at 10:00 hr. The minimum of exterior surface temperature was 20.7 °C and occured at 21:00 hr.

Table 2 showed dataset of space temperatures for green facades with 0%, 50%, and 90% leaves coverage area. The average of space temperatures on the experiment 1 with 0% leaves coverage area was 26.4 °C for indoor temperature and 31.7 °C for outdoor temperature. The maximum of indoor space temperature was 27.0 °C and occured at 16:00 hr and 20:00 hr. The minimum of indoor space temperature was 26.0 °C and occured from 23:00 hr to 05:00 hr. The maximum of outdoor space temperature was 33.7 °C and occured at 19:00 hr. The minimum of outdoor space temperature was 29.5 °C and occured at 06:00 hr.

The average of space temperatures on the experiment 2 with 50% leaves coverage area was 25.3 °C for indoor temperature and 25.7 °C for outdoor temperature. The maximum of indoor space temperature was 26.0 °C and occured from 11:00 hr to 15:00 hr. The minimum of indoor space temperature was 24.5 °C and occured at 06:00 hr. The maximum of outdoor space temperature was 30.0 °C and occured from 08:00 hr to 11:00 hr. The minimum of outdoor space temperature was 23.0 °C and occured at 23:00 hr, 03:00 hr, and 04:00 hr.

The average of space temperatures on the experiment 3 with 90% leaves coverage area was 24.7 °C for indoor temperature and 25.0 °C for outdoor temperature. The maximum of indoor space temperature was 25.1 °C and occured at 06:00 hr. The minimum of indoor space temperature was 24.2 °C and occured at 07:00 hr. The maximum of outdoor space temperature was 28.0 °C and occured from 23:00 hr to 00:00 hr. The minimum of outdoor space temperature was 23.0 °C and occured at 06:00 hr, 10:00 hr, 11:00 hr, 13:00 hr, and 14:00 hr.

Table 3 showed dataset of relative humidities for green facades with 0%, 50%, and 90% leaves coverage area. The average of relative humidities on the experiment 1 with 0% leaves coverage area was 66.1% for indoor relative humidities and 62.9% for outdoor relative humidities. The average of relative humidities on the experiment 2 with 50% leaves coverage area was 70.1% for indoor relative humidities and 69.0% for outdoor relative humidities. The average of relative humidities on the experiment 3 with 90% leaves coverage area was 72.5% for indoor relative humidities and 70.5% for outdoor relative humidities.

**Table 3:** Relative humidity dataset of experiment 1, 2, and 3 on 10th, 13th, and 16th December 2013.

Time	10/12/2013 (experiment 1)		13/12/2013 (experiment 2)		16/12/2013 (experiment 3)	
	HR_in_	HR_o_	HR_in_	HR_o_	HR_in_	HR_o_
6:00	65	60	70	62	75	70
7:00	66	63	68	65	70	65
8:00	65	60	68	65	70	65
9:00	67	60	65	65	68	65
10:00	62	57	65	63	68	65
11:00	60	55	63	60	65	64
12:00	60	50	63	60	65	63
13:00	60	50	63	65	65	65
14:00	58	54	63	65	65	66
15:00	58	55	63	65	65	70
16:00	60	60	65	70	70	70
17:00	60	65	65	70	70	72
18:00	65	70	70	73	73	73
19:00	65	65	70	75	75	75
20:00	65	70	73	75	75	75
21:00	70	70	75	75	75	75
22:00	70	70	75	75	80	75
23:00	70	70	75	75	80	75
00:00	73	70	78	72	80	75
1:00	73	70	78	72	80	75
2:00	75	70	78	73	78	75
3:00	75	65	80	75	78	75
4:00	75	65	75	70	75	75
5:00	70	65	75	70	75	70

HR_in_: indoor relative humidity; HR_o_: outdoor relative humidity.

## 2 Experimental design, materials, and methods

### 2.1 Description of building model

**Fig. 2:**
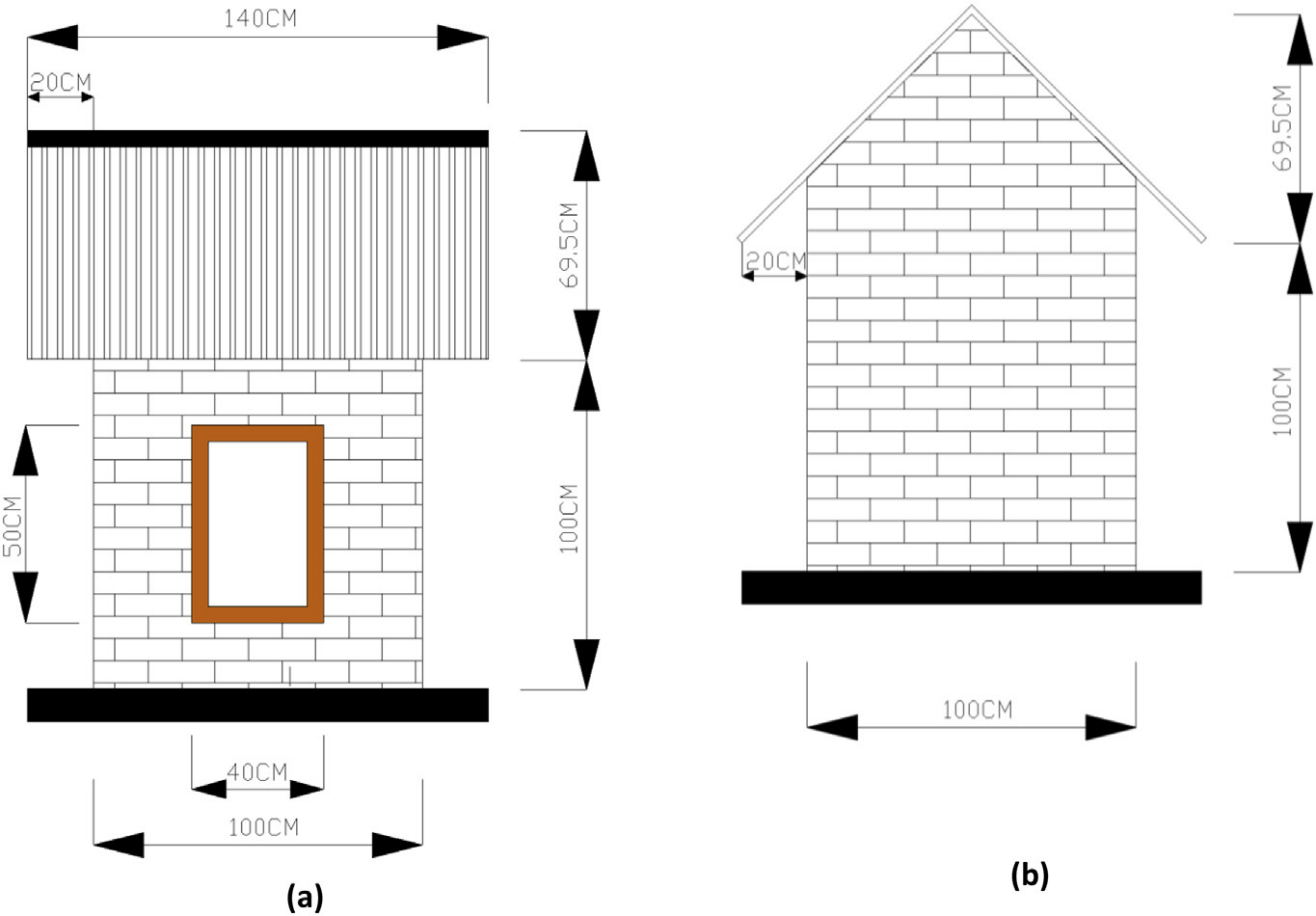
Detail of building model. **(a).** Front and back view; **(b).** Side views.

The building model located in Architecture Department of Diponegoro University in Semarang (Indonesia). The size of building model is 1 m × 1 m x 1 m or 1:4 from the real building. The walls were built from brick and the roof used tile roof. The ceiling was from asbestos and the floor used ceramic. The roof of the building model is gable roof. The model has two small windows as inlet and outlet allowed air circulation and heat flow inside. The model can be seen in Fig. 1 and the detail of building model size can be seen in Fig. 2.

**Fig. 3:**
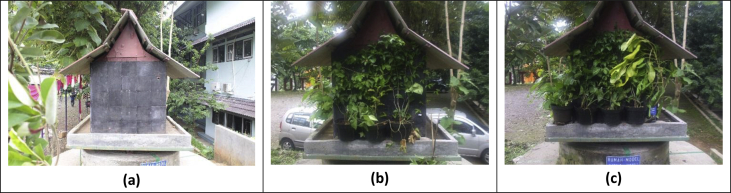
Model of green facade systems in experiment. **(a).** Green facade with 0% coverage area; **(b).** Green facade with 50% coverage area; **(c).** Green facade with 90% coverage area.

### 2.2 Description of green facade

The green facade systems was attached on the east facade of building model. *Passifloraflavicarva* and *Pseudocalymmaalliaceum* were types of climbing plants used as green facade. These species are local plants, evergreen, and have wide leaves surface.

In this experiment, the authors used direct green facade systems, can be seen in Fig. 3. The climbing plants were planted in the planter boxes and climbed directly on the wall of building model. Wire mesh was used as supporting structures. The green facade systems were divided into three types based on the percentage of leaves coverage area: 0% (without green facade); 50%; and 90%.

### 2.3 Field measurement

**Fig. 4:**
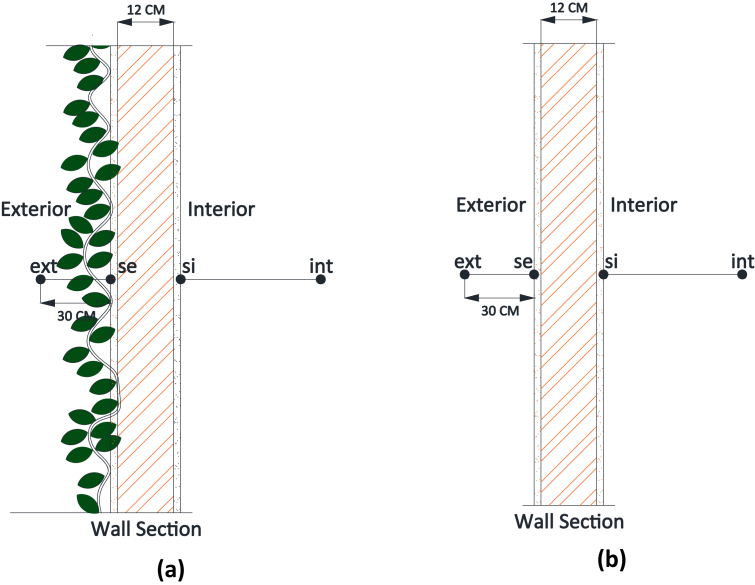
Illustration of data measurement for space temperatures and relative humidities. **(a).** Green facade section; **(b).** Bare wall section.

The green facades has been monitored using hygro thermometer and infrared thermometer. Hygro thermometer was used to measure space temperatures and relative humidities. It was placed at a distance 30 cm from facade for outdoor space temperatures and outdoor relative humidities, can be seen in Fig. 4. As for indoor temperatures and indoor relative humidities, hygro thermometer was positioned in the middle of building model. The accuracy of hygro thermometer is stated ±1 °C with temperature range from −10 °C to 60 °C.

**Fig. 5:**
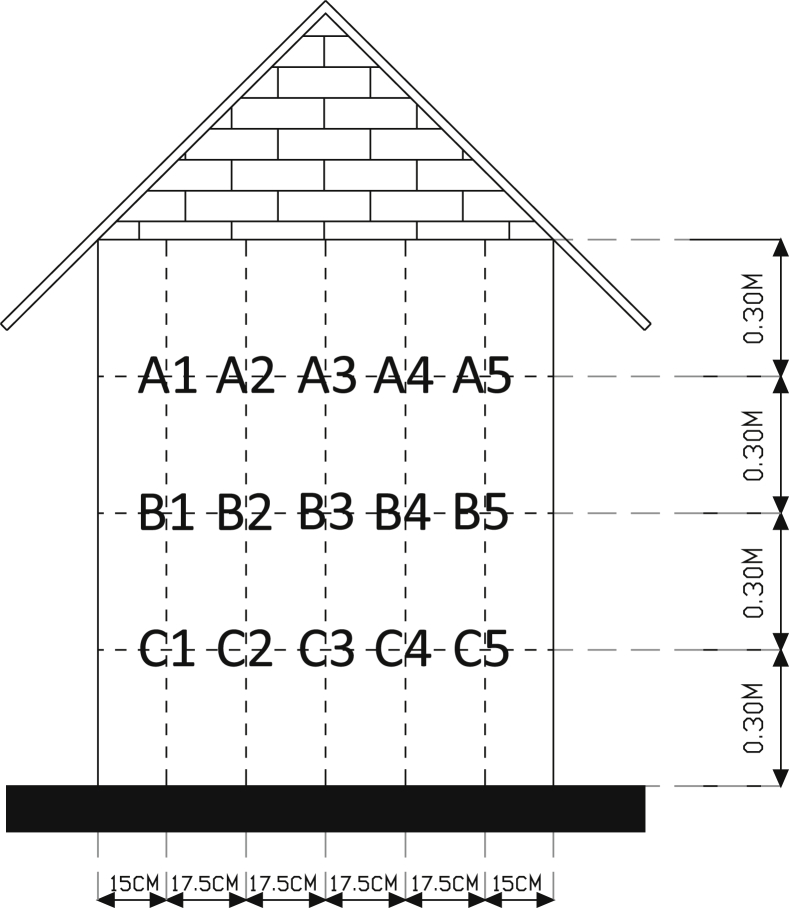
Illustration of measurement points for surface temperature data collection.

Infrared thermometer was used to measure surface temperatures. It was directed on specific measurement point on the facade surface to collect dataset of surface temperatures. The accuracy was ±2% with temperature range from −50 °C to 280 °C. Dataset of surface temperatures were collected through divided facade surface into fifteen measurement points, can be seen Fig. 5. They are five on the top, five on the middle, and five on the bottom. The difference in each measurement point was from 0 °C to 0.1 °C. The dataset of surface temperatures are the average of fifteen measurement points. Local weather conditions were collected from Maritime Meteorology Station of Semarang City [2]. All dataset were acquired at 1 (one) hour interval.

## Funding sources

The dataset is part of a research project entitled The Effect of Leaves Density on Vertical Garden to Provide Indoor Thermal Comfort financed by LPDP scholarship (Indonesia Endowment Fund for Education by Indonesian Ministry of Finance), in the batch program V in 2014, under the register number 0038543/TK/T/3/lpdp2014. The authors also fully acknowledge the grant given by Universiti Brunei Darussalam.
